# How does mobility and urban environment affect the migrants’ settlement intention? A perspective from the intergenerational differences

**DOI:** 10.3389/fpubh.2024.1343300

**Published:** 2024-03-01

**Authors:** Xiaoxiang Liang, Qingyin Li, Wen Zuo, Rong Wu

**Affiliations:** ^1^Baiyun Branch of Guangzhou Urban Planning & Design Survey Research Institute, Guangzhou, China; ^2^School of Architecture and Urban Planning, Guangdong University of Technology, Guangzhou, China

**Keywords:** migrants, settlement intention, new generation, urban environment, mobility

## Abstract

**Introduction:**

With China embracing a new people-centered urbanization stage, the problem of migrants “flowing without moving” has become increasingly prominent, and settlement intention has gradually garnered attention.

**Methods:**

Our research, based on questionnaire data from the China Labor Force Dynamic Survey 2016, uses a multilevel linear regression model to explore the influence of mobility, social environment, built environment, and demographics characteristics on settlement intention in the migrants and discusses differences between settlement intention of new and old generations and their internal influence mechanism.

**Results:**

The findings are as follows: (1) Compared to the old generation, the new migrant generation generally has higher settlement intention. (2) The migrants’ settlement intention is influenced mainly by mobility, social environment, built environment, and demographic characteristics. (3) For the new migrant generation, social and demographic characteristics significantly influence their settlement intention. (4) The floating and built environment of the old generation significantly influence their settlement intention.

**Discussion:**

Finally, this paper argues that there are differences in the influence mechanism of the same factors on the settlement intention of the new and old generations of migrants. It proposes differentiated policy suggestions for the migrants to promote city social integration. Finally, this paper argues that there are differences in the influence mechanism of the same factors on the settlement intention of the new and old generations of migrants. It proposes differentiated policy suggestions for the migrants to promote city social integration.

## 1 Introduction

Migrants have made important contributions to the countries and communities of origin and destination. How to promote the integration and residence of migrants has become a particularly complex topic, which has been studied by scholars all over the world (1). Since its reform and opening up, China’s urbanization has developed rapidly and is now in a critical period of transition from high speed to high quality. The migrants, that is, those who have lived outside the household registration area for more than six months in China, are an important factor in promoting China’s social development (2). However, owing to the household registration system and other reasons, the migrants cannot enjoy the same public services and social security as local people (3), and some migrants are in a weak and marginalized position in cities and in relation to finding work (4). “Cities that cannot be integrated, villages that cannot be returned” has become an accurate portrayal of many migrants. Therefore, the migrants’ settlement intention in cities has become an important factor affecting China’s new people-oriented urbanization strategy. China’s “14th Five-Year Plan” clearly indicates that people should enjoy a higher quality of urban life. In this context, studying the migrants’ settlement intention is of great significance, as it can provide a decision-making reference for improving the migrant management system in cities and promoting their integration into cities.

Western scholars have conducted many theoretical discussions on the factors influencing population mobility and migration decision making, such as push-pull theory (5), neoclassical models (5, 6), the theory of labor market segmentation (7), behavioral methods (8, 9), etc. With the deepening of globalization and the decline and reform of the welfare state, immigration has become increasingly common. New migrants are no longer refugees and poor people as in the traditional view, but may be high-quality technical talents and entrepreneurs who have better prospects in the labor market and are more likely to enter the upper class (6). In the context of the Western capitalist market, economic, social, and cultural factors are often emphasized, while institutional factors are ignored. However, China’s population mobility is also influenced by the household registration system, land policy, and social security (7–10).

At present, Chinese scholars’ discussions of the factors influencing the migrants’ settlement intention can be divided into four aspects: individual, family, economy, and society (11, 12). Early research regarded the household registration system as the main factor hindering migrants from settling in cities (8). However, with reform of the household registration system, the influence of household registration on the migrants has declined, and the market system has replaced it (13). At the same time, the migrants’ settlement intention is not only affected by economic and social factors but also by the characteristics of flowing cities (14). Intergenerational differences have become a popular topic in the study of migrants in recent years. Intergenerational differences have been identified between the new and old generations of the migrants in life experience, local identity, motivation to go out, social integration, and settlement intention (15).

In summary, existing studies have the following shortcomings: (1) Previous studies have mostly analyzed the impact of social and cultural factors on the migrants’ settlement intention from the perspective of sociology, and lack attention to urban built environment. Therefore, this study adds urban spatial elements and discusses their impact on the migrants’ settlement intention. (2) Previous studies have paid less attention to inter-generational differences between the new and old generations. This study compares the new and old generations of the migrants to analyze the differences in the influencing factors and mechanisms of their settlement intention. (3) In the context of new urbanization, urban agglomerations will become the main form of urbanization in China (16). On this basis, this paper takes the Pearl River Delta region as an example to explore the influencing factors and mechanisms of migrants’ settlement intention from three dimensions: mobility, demographics characteristics and urban environment. Differences in settlement intention can provides a reference for exploring the high-quality development of cities.

## 2 Literature review

### 2.1 Settlement intention and its measurement

Previous studies have widely discussed willingness to settle as an important factor of the migrants’ subjective feelings. Data acquisition is mainly through the CMDS (China Migrants Dynamic Survey) national migrants data monitoring platform, social research, interviews and other ways, and sampling surveys, and other ways to screen samples for research. Generally, the relevant discussions are conducted through the migrants’ demographic, social environment, mobility, and built environment (17). Among them, in terms of demographic characteristics, factors including gender, age, marriage, education level, and so on are selected for discussion. In the aspect of social environment, including household registration, income level, cultural identity, and other factors, the influence mechanism behind settlement intention is explored through the study of the social economy and culture of the migrants. The flow characteristics include its reasons, the number of people moving with them, and others, which are studied according to the migrants’ flow experience. In terms of built environment, the city’s built-up environment, including urbanization level, per capita GDP, public services, and other factors, also has an impact on settlement intention. Using mathematical models can quantify research indicators quickly, intuitively, conveniently, and accurately and make research results more rational and reliable, so as to infer the influence of various factors on the migrants’ living intentions. For example, Han et al. used a probability model and binary logic model to analyze the migrants of ethnic minorities in many aspects, to explore the influencing factors of their settlement intention, and to draw a conclusion that social and psychological interactions have a significant positive impact on such settlement intention (17).

In addition, with the expansion in the scope of discussion and the deepening of the research, the research direction and model methods of the factors influencing the migrants’ settlement intention have become more diverse. For example, to study the influence of the action of creating a civilized city on settlement intention, Guo et al. found that this creation process inhibited the migrants’ settlement intention. Research has shown that the settlement intention of the high-quality migrants has not been enhanced, but the low-quality migrants will reduce their settlement intention in the short term due to the rigid constraints of the city’s appearance and income reduction (18). Yue et al. used satellite remote sensing data of PM2.5 concentration in various cities to further test the relationship between subjective air pollution and migrants’ interest in settling down (19). In general, the more diverse research directions and data collection methods in the present era provide more research angles and methods to study the factors influencing the migrants’ settlement intention, allowing us to discuss their influence on settlement intention.

### 2.2 Determinants of migrants’ settlement intention

Previous studies have discussed the factors that affect the migrants’ settlement intention in many aspects, mainly focusing on the demographic, social environment, mobility, and built environment of such population. First, in terms of demographic characteristics, previous discussions have focused on the influence of the intrinsic characteristics of the migrants on the settlement intention, among which gender and education level are considered important influencing factors. Studies have shown that women are more willing to settle than men, which is related to their more staged migration (20). In addition, the education level has been proven to be related to human capital and to have a positive impact on the migrants’ settlement intention (21). Second, in terms of social environment, several studies have found that economic factors, household registration systems, and social culture have a substantial impact on migrants. The higher the income level of the migrants in the local area, the lower the cost of living, and the more the settlement of the migrants can be promoted (22). Many discussions in early studies have addressed household registration systems. Taking China as an example, Wang et al. found that its urban well-being policy is linked to the household registration system, and the difficulty of settling restrictions and the well-being enjoyed by household registration play an important role in the migrants’ willingness to settle (8, 23). At the same time, social culture mainly influences the migrants’ settlement intention through psychological identity, local attachment, and social integration (17, 23). The research shows that social psychological integration at the local level and the local attachment of the migrants have a positive impact on shaping its settlement intention (17, 24–27). For minority groups, identity is extremely important, and the migrants tend to stay in cities with higher cultural homogeneity (17).

In addition, in terms of mobility, family is an important factor affecting the migrants’ settlement intention. Many studies have shown that different migration patterns affect such willingness and that migration with family members can improve the migrants’ emotional sense of belonging. Parents with children moving with them are more willing to permanently settle in cities and towns compared to parents without children moving with them (28). In addition, in terms of built environment, Le et al. found that urban population density and housing prices have a significant U-shaped effect on settlement intention, which is one of the main factors affecting the settlement intention of the long-term migrants (29). Tan et al. also found that an inclusive and friendly living environment and an open and diversified housing market affect the migrants’ settlement intention (10). It is worth noting that the related research on air pollution also shows that health factors play an important role in the migrants’ willingness to settle down (19, 30–33). In general, the factors that affect such willingness are rich and varied, but the economic and cultural factors in social environment have an impact on it.

### 2.3 Intergeneration difference in settlement intention

The theory of intergenerational difference was proposed by German sociologist Mannheim in the 1950s, which defined “generation” as an identifiable group comprising individuals with common birth age, age stage, key growth stage, and major life events and emphasized its social and cultural characteristics (34). The difference in social experience and values between the new and old generations will affect their concern about settlement intention, which has led to academic research on its influencing factors. Studies have shown differences and the same influencing factors of settlement intention between the two generations. Although differences exist in the details of settlement intention between the two generations, no fundamental difference has been observed, and the new generation’s settlement intention is not stronger than that of the old generation (35). However, Tang and Feng draw the opposite conclusion, namely that the new generation of migrants is more willing to settle in existing cities- especially big cities. The new generation’s settlement intention is more significantly influenced by geographical and socio-economic characteristics, values its own development, obtains better experience opportunities, and has a stronger localization trend. Conversely, the old generation pays more attention to family factors and tends to live in stable conditions (36).

At the same time, attention has been paid to the influence of the different backgrounds of the two generations on settlement intention. The two generations of migrants holding land have different settlement intention in cities, and the migrants, with important assets and emotional reasons such as farmland and homestead in their hometown, have lower willingness to stay (37, 38). The equalization of farmland ownership between generations will gradually reduce the gap in rural migrants’ willingness to settle in China (37). Few studies have addressed intergenerational differences in the settlement intention of the two generations of migrants, and not enough studies have focused on the influencing factors of settlement intention between the two generations with different values. Thus, it is necessary to further explore the influencing factors of settlement intention between generations and provide suggestions for promoting high-quality urbanization development.

## 3 Research design

### 3.1 Case location and data source

**Figure 1:**
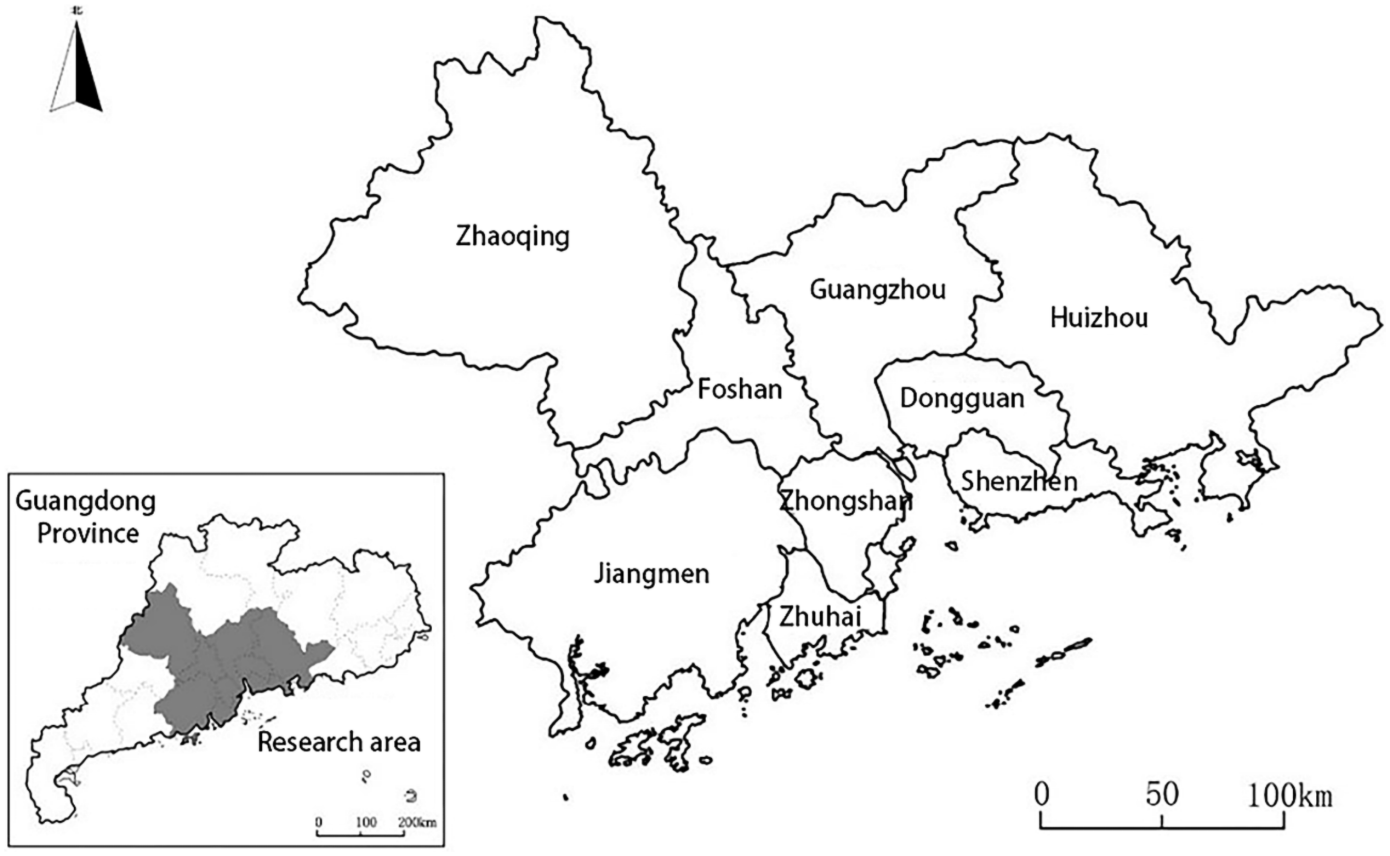
Location of the Pearl River Delta region.

The data used in this study originate from the China Labor-force Dynamics Survey (CLDS) 2016, which provides a tracking database at the individual, household, and community levels (available online at http://css.sysu.edu.cn/Data, accessed on May 1, 2019). The CLDS, a biannual follow-up survey of village dwellings and rural areas in China, was conducted by the Center for Social Survey of Sun Yat-sen University. It established a comprehensive database of labor based on demographic characteristics, socioeconomics, housing conditions, and community contexts in the survey, which is still used in many studies today (39–41). The Pearl River Delta is a region with a high level of economic development in China, which can provide a large number of employment opportunities and attract a large number of migrants. It is a representative city for the citizenization of migrants in China, so the Pearl River Delta region is chosen as the research site (21). We selected the sample data from nine cities (Figure 1): Guangzhou, Foshan, Zhaoqing, Shenzhen, Dongguan, Huizhou, Zhuhai, Zhongshan, and Jiangmen. The “migrants” of this study is defined as people who have lived outside the household registration place for more than six months, and these people mainly migrate within China. A total of 483 valid samples were obtained after screening. The CLDS-2016 questionnaire used in this study included information on workers’ backgrounds, educational experience, migration history, social participation and support, employment status, and health status.

### 3.2 Population attribute characteristics

According to data from the Seventh Population Census, the population growth of Pearl River Delta city clusters has accelerated, and the degree of agglomeration has increased, with Shenzhen, Guangzhou, Foshan, and Dongguan showing continued attraction to the migrants. This paper defines the new generation of migrants as “the migrants born in 1980 and later,” referred to as “the new generation,” and “the migrants born before 1980 has become the old generation” referred to as “the old generation” (36, 42, 43). The total number of samples counted was 483, of which 213 were new generation and 270 were old generation samples. The average age of the total sample was 38 years old, the male to female gender ratio is 44.31:55.69, married persons accounted for 86.75% of the total sample, whereas local households accounted for only 4.76% of the total sample. In terms of educational attainment, bachelor’s degree (college) and above accounted for only 5.59% of the total sample, which is a low level of education, whereas the proportion of party members was 3.11%. In terms of self-assessed health, the proportion of those who rated themselves as “healthy” was the largest, at 44.10%. The proportion of those with an annual household income of 25,000 to 50,000 RMB was 31.88% and the proportion of those with an annual household income of 50,000 to 100,000 RMB was 37.47%.

Table 1 shows the differences in the demographic characteristics of the new and old generations. The proportion of females in the new generation (61.50%) is larger than that of the old generation (51.11%); the old generation is mostly married, while the proportion of the new generation who are unmarried, divorced, or widowed (43.37%) is larger in relation to the old generation; the proportion of party members in the new generation (5.16%) is higher than that of the old generation (1.48%); in terms of educational attainment, the proportion of the new generation with a bachelor’s degree (junior college) and above was 11.74%, while the proportion of the old generation was only 0.74%; in terms of self-assessed health, the proportion of the new generation whose self-assessed health was “healthy” (51.64%) is larger than that of the old generation (38.15); in terms of total annual household income, both the new and old generation are in the range of 50,000–100,000 yuan, but in terms of 100,000–200,000 yuan of total annual household income, the proportion of the new generation (17.37%) is higher than that of the old generation (13.70%). The education level, proportion of party members, and income level of the new generation are higher than those of the old generation.

**Table 1:** Demographics characteristics of the sample.

Characteristics	Total	Old generation	New generation
Number of samples	483	270	213
Age mean/S.E. (years)	38.48/10.61	46.41/6.34	28.54/5.04
Gender (%)			
Male	44.31	48.89	38.50
Female	55.69	51.11	61.50
Education (%)			
Primary school and below	26.29	40.37	8.45
Middle and high school	68.12	58.89	79.81
College and above	5.59	0.74	11.74
Hukou status (%)			
Local	4.76	4.60	4.98
Nonlocal	95.24	95.40	95.02
Political affiliation (%)			
Member of the communist party	3.11	1.48	5.16
Non-communist party member	96.89	98.52	94.84
Marital status (%)			
Married	86.75	97.41	73.24
Unmarried/divorced/widowed	13.25	2.59	26.76
Self-assessed health status (%)			
Very unhealthy	0.41	0.74	0
Quite unhealthy	6.00	9.26	1.88
Normal	29.81	33.70	24.88
Healthy	44.10	38.15	51.64
Very healthy	19.67	18.15	21.60
Family annual income (%)			
0-25,000 RMB	12.63	17.41	6.57
25,000–50,000 RMB	31.88	28.52	36.15
50,000–100,000 RMB	37.47	37.78	37.09
100,000–200,000 RMB	15.32	13.70	17.37
200,000 RMB and above	2.69	2.59	2.82

### 3.3 Variable selection and measurement

**Figure 2:**
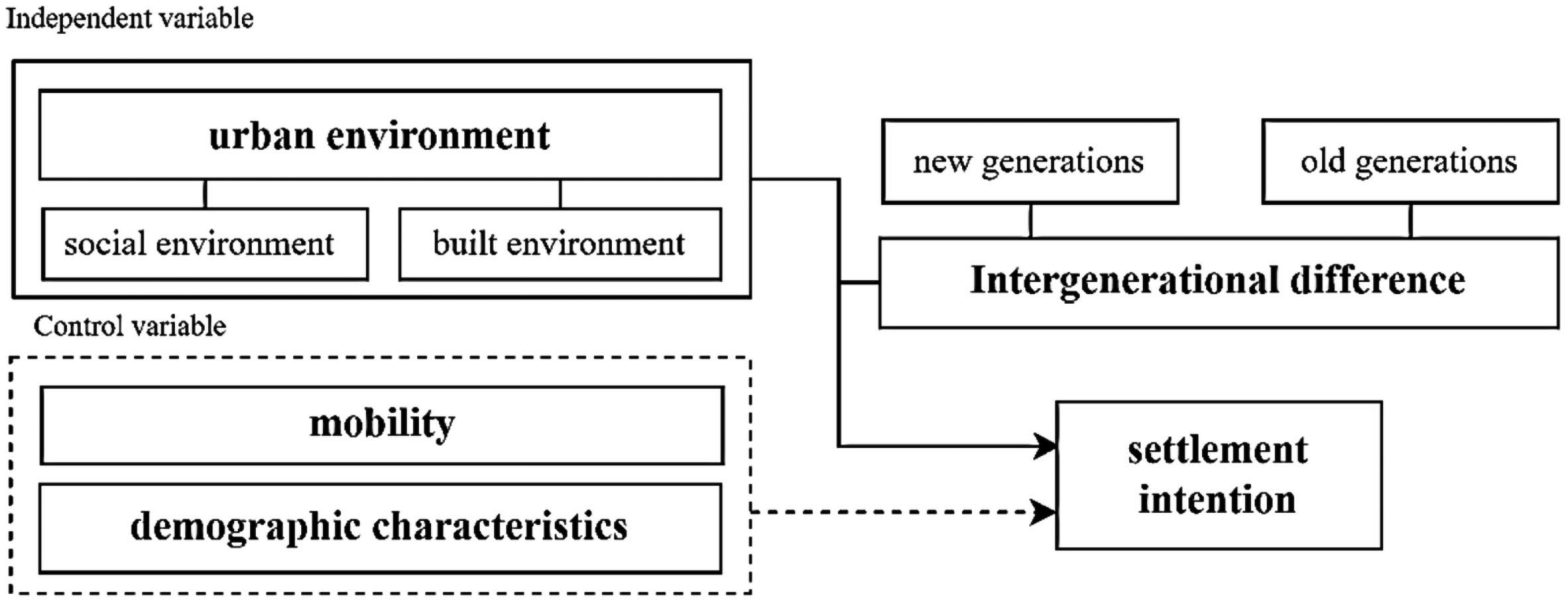
Conceptual framework.

According to relevant literature, we selected three dimensions that affect migrants’ settlement intention: mobility, demographic characteristics, and urban environment, among which urban environment includes social environment and built environment (Figure 2).

#### 3.3.1 Settlement intention of migrants

The dependent variable in this paper is the settlement intention of the migrants, which is measured by the question, “Are you likely to settle in local in the future?” The responses measure the strength of the willingness of the migrant population to stay in the local area for the long term, with the scores ranging from 1 (“very unlikely”), 2 (“rather unlikely”), 3 (“uncertain”), 4 (“rather likely”), and 5 (“very likely”).

#### 3.3.2 Mobility

According to existing research, mobility affect the settlement intention of the migrants (17). The longer the migrants move outside, the wider the scope and the stronger their settlement intention in the inflow area. Therefore, the variables selected in this study include whether parents have migrant experience, the number of migrations, and reasons for migration. Reasons for migration were grouped into three categories: work and study, marriage and relocation, and demolition and moving.

#### 3.3.3 Urban environment

Specifically, we divided the urban environment into built environment and social environment. In terms of social environment, according to previous studies, social integration means that the immigrant population gradually accepts and adapts to the social culture of the immigrant location, and thus develops benign interactive communication (44). In addition to their economic aspirations, migrant populations aspire to establish wider social networks in the inflow area and hope to feel safe and comfortable in the social environment in the inflow area. Therefore, this study selected the number of friends, community trust, community safety, community participation, and other variables to analyze. Community trust is measured on a 5-point scale, ranging from 1 point to 5 points, which means “very high probability” to “very low probability.” The higher the score, the stronger the sense of community security. The overall score on the scale ranges from 6 to 30 points. The Community Participation Scale has a total of 9 indicators, and also adopts a 5-point scale, with scores ranging from 1 to 5, from “never participate” to “participate every day,” with higher scores indicating better participation in organizational activities, and with an overall range of scores from 9 to 45. Through the reliability test, the α value of the Community Safety Scale is 0.704, which indicates high reliability. The reliability of the Community Participation Scale was 0.525 (Table 2).

**Table 2:** Dimensions of community safety and participation in organizational activities.

	Explanation of variables
Community safety	Likelihood of experiencing unemployment in the next five years
	Likelihood of experiencing crime in the next five years
	Likelihood of experiencing a terrorist attack in the next five years
	Likelihood of consuming fake medicines or shoddy food in the next five years
	Likelihood of being exposed to an infectious disease in the next five years
	Likelihood of experiencing environmental pollution problems in the next five years
Community participation	Frequency of participation in the activities of neighborhood committees
	Frequency of participation in activities of social work organizations
	Frequency of participation in activities of owners' committees
	Frequency of participating in activities of leisure/entertainment/sports clubs/ salons, etc.
	Frequency of participation in activities of learning/training organizations
	Participation in activities of hometown associations
	Participation in activities of clansmen organizations
	Participation in activities of public welfare/social organizations/volunteer groups
	Frequency of participating in activities of religious organizations

The built environment of the inflow locations, including their development level, environment, and public service facilities, has become the focus of the migrants (8). Therefore, the urbanization rate, population density, land use intensity, GDP *per capita*, green space coverage, number of hospitals, number of POI, and annual average concentration of PM2.5 of the nine PRD cities were selected to measure the built environment of the cities in this paper (Table 3). Among them, the data used for urbanization rate, population density, land use intensity, GDP *per capita*, greenery coverage, number of hospitals, and number of POI were obtained from the 2016 statistical yearbooks, the statistical bulletin on national economic and social development (45), and the data on the average annual concentration of fine particulate matter (PM2.5) were obtained from the “Ranking of PM2.5 Concentration of 366 Cities in China in 2016” (46). The data used are objective, comprehensive, and highly authoritative.

**Table 3:** Summary statistics of the sampled cities.

	Guang zhou	Foshan	Zhaoqing	Shenzhen	Dong guan	Hui zhou	Zhuhai	Zhong shan	Jiang men
Urbanization rate (%)	86.06	94.95	46.08	100	89.14	69.05	88.80	88.20	65.06
Population density (persons/km^2^)	1897.77	1240.4	274.30	5689.45	3358.29	421.00	917.76	1761.38	478.06
Number of POI (10,000 persons/unit)	817.59	813.52	365.08	1041.16	1175.15	645.12	939.95	1238.42	508.22
Land use intensity (%)	16.80	4.18	0.80	46.22	40.08	2.31	8.14	7.80	1.65
GDP per capita (yuan)	141933	115891	51178	167411	82682	71605	134548	99471	53374
Green space coverage (%)	41.80	40.96	36.51	45.11	47.67	42.94	47.74	38.20	44.08
Annual average Concentration of PM2.5 (μg/m^3^)	36.1	38.5	37.5	27.1	35.3	26.9	26	30.1	33.9
Number of hospitals	273	115	148	136	89	144	53	53	41

### 3.4 Data analysis

The data analysis method used in this paper is the multilayer linear regression model, compared with the traditional statistical methods, the multilayer linear regression model can distinguish the impact of different levels on the explained variables, the assumptions in the model are more in line with the actual situation, and the results obtained can be more reasonable and correctly reveal the real relationship between things. The multilayer linear regression model in this paper is divided into two layers, which is based on the methodology mainly proposed by Joop Hox (47):


$$Y_{ij}=\alpha_{1}+\overset{n}{\underset{\begin{array}{c} i=1\\ j=1 \end{array}}{\sum }}\beta_{ij}Z_{ij}+\overset{n}{\underset{j=1}{\sum }}\gamma_{j}W_{j}+\mu_{ij}+\varepsilon_{j}$$

where: Y_ij_ represents the settlement intention of the floating population; α_1_ represents the intercept; Z_ij_ represents the individual level variable of i sample in j city, and β_ij_ represents the regression coefficient of i sample in j city; W_j_ represents the city-level variable of j city; γ_j_ represents the regression coefficient of j urban variable; μ_ij_ is the error term at the individual level of the sample i in j city, and ε_j_ is the error term at the city level.

In this study, the suitability of a multilayer linear model was determined based on the intragroup correlation coefficient (ICC) of the null model (48).


$$ICC=\frac{\sigma_{b}^{2}}{\sigma_{w}^{2}+\sigma_{b}^{2}}$$

Where $\sigma_{b}^{2}$ represents the intergenerational variance; $\sigma_{w}^{2}$ represents the individual variance of the urban migrant population. According to the calculation results of Stata, the ICC value is as large as 0.14, which indicates that there are differences in the settlement intention of the migrants in different generations; therefore, it is necessary to set up a multilayer model to analyze the data.

## 4 Settlement intention of the migrants

In this study, five factors, namely, generation type, household registration type, political background, marital status, and parents’ mobility experience, were selected for the independent sample *t*-test (Table 4). The results showed that the *p*-value of generation type and parents’ mobility experience was significantly different less than 0.01, and the result was significant. In terms of generation types, the average settlement intentions of the old generation and the new generation are 2.21 and 2.56, respectively. The settlement intentions of the new generation are stronger than those of the old generation, which is due to the former’s lack of social experience, and their more urgent need to form a stable lifestyle to enhance their sense of stability and self-confidence. The migrants whose parents had floating experience had a higher settlement intention. Because they have similar family backgrounds, they have a deeper feeling of instability brought about by population mobility, which makes them want to break away from it. Moreover, parents’ floating experience can provide them with certain social experiences, which are helpful for the migrants in realizing their settlement needs (49). The differences between household registration type, political background, and marital status were not significant (all *p* > 0.05). In terms of household registration type, the settlement intention of the non-agricultural registered permanent residence migrants is higher than that of the agricultural household registration migrants; in terms of marital status, the settlement intention of the unmarried, divorced, and widowed migrants is higher than that of the married migrants, which is contrary to existing research (3, 50), and may be related to loneliness caused by a lack of marital companionship.

**Table 4:** Settlement intention of floating migrants in Pearl River Delta.

Demographics characteristics		Settlement intention		
		Mean	*T-*value	*P-*value
Generational type	Old generation (before 1980)	2.21	−2.727	0.007*
	New generation (after 1980)	2.56		
Hukou status	agriculture	2.35	−0.996	0.320
	non-agricultural	2.68		
Political affiliation	Member of the communist party	2.35	−1.203	0.230
	Non-communist party member	2.80		
Marital status	Unmarried	2.56	1.381	0.171
	Married	2.34		
Parental migrant experience	No	2.30	−2.013	0.045*
	Yes	2.62		
Settlement intention	–	2.37		

***p < 0.01, **p < 0.05, *p < 0.1.

## 5 Mechanism analysis of settlement intention

Based on the multilayer linear model proposed in the previous section, we analyzed the mechanism of influence of demographics characteristics, migrant, social environment, and built environment of the migrants in the Pearl River Delta region on their settlement intention, and compared the two sub-samples to analyze the differences in the mechanism of influence of the settlement intention of the new and old generations.

### 5.1 Analysis of total sample results

Several factors affect the migrants’ settlement intention. Table 5 presents the results of the model analysis for the entire sample. In terms of built environment, urbanization rate, population density, land use, green coverage rate, and settlement intention were significantly related. There was a significant negative correlation between urbanization rate and settlement intention; that is, the higher the urbanization rate, the lower the settlement intention. The urbanization rate is usually used as one of the standards to measure urban development, which mainly affects people’s settlement intentions through implied aspects such as household registration status, public services, and infrastructure. The higher the urbanization rate, the more developed the city. The higher cost of living and higher level of social exclusion in big cities make it difficult for the migrants to live there long-term, thus reducing their settlement intention (3). Population density has a positive impact on settlement intention; that is, the denser the population density, the stronger the settlement intention. This result is consistent with the relevant research conclusions (29). Higher population density indicates that the area has economic, political, and ecological advantages, which can bring corresponding benefits to the local population and attract population agglomeration (51).

**Table 5:** Model for floating migrants’ settlement intention of the sample.

	Total sample		
	Coefficient	Standard error	*P*-value
Demographics characteristics			
Gender (reference group: female)			
Male	−0.100	0.121	0.411
Education level (reference group: primary school and below)			
Junior high school, senior high school, and technical secondary school	0.097	0.140	0.489
Bachelor degree or above	1.299***	0.287	0.000
Father's household registration type (reference group: agricultural registered permanent residence)			
Non-agricultural registered permanent residence	0.107	0.295	0.715
Political outlook (reference group: non-party members)			
Party member	0.029	0.337	0.931
Marital status (reference group: unmarried/divorced/widowed)			
Married	−0.077	0.184	0.674
Number of family members	0.126***	0.032	0.000
Self-rated health status	−0.092	0.070	0.189
Annual household income	0.000**	0.000	0.004
Mobility			
Number of migrations	−0.118**	0.043	0.006
Parents' mobility experience (reference group: none)			
Yes	0.195	0.146	0.182
Reasons for mobility (reference group: work-study flow)			
Matrimonial migration	0.587**	0.209	0.005
Demolition and moving flow	−0.223	0.401	0.578
Urban environment (urban built environment and social environment)			
Social environment			
Number of friends	0.013*	0.006	0.040
Community trust degree	0.050	0.069	0.471
Participation in community organization activities	0.025	0.058	0.661
Community security	−0.006	0.014	0.792
built environment			
Urbanization rate	−8.094*	3.188	0.011
Population density	0.001*	0.000	0.037
POI number per 10,000 people	0.001	0.001	0.113
Land use situation	−9.307*	3.711	0.012
Per capita GDP	0.000	0.000	0.203
Green coverage rate	14.933*	6.680	0.025
Annual average concentration of PM2.5	0.053	0.045	0.238
Number of hospitals and health clinics	0.000	0.002	0.803
Constant	−1.332	2.684	0.620

***p < 0.01, **p < 0.05, *p < 0.1.

In addition, the gathering of migrants with similar cultural backgrounds can promote the development of cultural identity, enabling the migrants to better adapt to the local area, and help improve their settlement intention (4, 17, 25). There was a significant negative correlation between land use and settlement intention, that is, the higher the land use intensity, the lower the settlement intention of the migrants. High-intensity land use competes with urban leisure and urban ecological spaces, reduces residents’ living comfort, and adversely affects their mental health (52). At the same time, higher land-use intensity reduces the leisure space for citizens’ communication, reduces the diversification of citizens’ lives, affects the promotion of urban vitality, and is not conducive to the formation of a good urban atmosphere; thus, the migrants’ settlement intention is reduced (53–55). Finally, there is a significant positive correlation between the green coverage rate and settlement intention; that is, the higher the regional green coverage rate, the higher the attraction for the migrants to settle down. The green coverage rate is related to the ecological environmental quality of the region. A higher green coverage rate is helpful for improving the absorption of carbon dioxide in cities and alleviating the impact of environmental pollution on people’s health (56). At the same time, it is helpful to improve the urban thermal environment, alleviate the urban heat island effect (57), promote the quality of the living environment, benefit the physical and mental health of local residents, and promote the migrants’ settlement intention.

In terms of social and demographic characteristics, higher education level, number of family members, and annual family income had a significant influence on settlement intention. Education is closely related to settlement intention. The higher the education level, the stronger the settlement intention. Education is an important way of improving human capital. Taking primary school and below as the reference group, junior college education level and above was positively correlated with settlement intention. This shows that higher education level helps the migrants to stay in the local area, which is consistent with existing research conclusions (3). Higher education means that migrants have richer human capital, are more likely to obtain better jobs and incomes, and are more psychologically confident, leading to an increase in their settlement intention. Simultaneously, the number of family members positively influenced settlement intention (10, 20). The increase of family members will enhance the willingness of floating population to settle down, which is consistent with existing research results (28, 50, 58), indicating that the more family members there are, the higher the immigration cost and the higher the intention to settle in the local area. In addition, families provide psychological support to the migrants, and the process of familization within the migrants increases the likelihood of them staying in the local area (50). The annual income of families has a positive orientation toward settlement intention, and with an increase in income, the settlement intention of the migrants increases. The higher the annual income of a family, the more resources and benefits that family members can obtain in the local area. This plays a positive role in promoting quality of life, improving comfort, obtaining sufficient self-confidence and security in life, and improving settlement intention to a certain extent (28).

Among the three indicators of immigration characteristics, the number of migrations is negatively correlated with settlement intention; that is, the greater the number of migrations, the lower the settlement intention. The influence of the migration time of the migrants on settlement intention was probably related to the stay time of the migrants in the local area (50). Related research shows a positive correlation between stay time and local attachment and that longer stays or communication are usually accompanied by stronger local attachment. Promoting local attachment can increase a population’s settlement intention in a local area (4, 52). In addition, the stay time was related to settlement intention. The longer the migrants stays in the local area, the better it adapts to it (58). The greater the number of migrations, the shorter the population stay time in the region. Therefore, shorter stay time males it difficult to cultivate a migrants’ sense of local ties, local attachment, and belonging, resulting in low adaptability to the regional environment, making the migrants less willing to stay there. There was a significant positive correlation between reasons for immigration and settlement intention accompanied by marriage. In terms of mobility reasons, compared with work-study mobility as a reference group, family members can share the cost of living, provide psychological support, reduce the cost of remigration, and help improve the settlement intention of the migrants (28). At the same time, among the four indicators of social environment, number of friends has a positive impact on settlement intention; that is, the more friends in the local area, the stronger the intention to stay. Obtaining more friends is conducive to forming a richer social network and promoting the social integration of the migrants in the local area, which can ensure more social relations and psychological comfort for the migrants (59). A migrants with richer social relations with local residents can obtain more useful information and practical support; therefore, they show a significantly higher settlement intention (60).

### 5.2 The contrast between the new and old generations

The model analysis results for the two-component samples from the new and old generations are shown in Table 6. Comparing the two-component samples, there are differences in the influence mechanism of different factors on the new and old generations. Compared to the new generation, the old generation pays more attention to the built environment of the immigration site, and they have a clearer purpose for migration. They should consider the management of the entire family, and the per-capita GDP of the place of immigration is one of the reasons for deciding whether to stay. At the same time, owing to the decline in physical function, the old generation often pays more attention to health management and is more sensitive to environmental quality. Green spaces can promote social interaction and reduce the impact of social isolation on old generations, which is very important for their health and well-being, and can help them mitigate the risk of disease (61). Therefore, compared with the new generation, the greenspace coverage rate has a much greater impact on the settlement intention of the old generation. In addition, the old generation was more inclined to settle in areas with lower urbanization rates, that is, the higher the urbanization rate, the lower their settlement intention. Areas with higher levels of urbanization are often accompanied by higher levels of environmental pollution, higher costs of living, faster pace of life, higher housing prices, and generally lower quality of living environments (62). The old generation has a higher demand for environmental livability (63), and its resistance to the problems brought about by high-level urbanization is more obvious. Therefore, the old generation pays more attention to the intensity of land use, which had a significant negative correlation with their settlement intention. The increase in land use intensity has adverse effects on the urban ecological environment and crowds out social open spaces (55), which reduces the quality of the living environment of the old generation in this area and their settlement intention.

**Table 6:** Models for floating migrants’ settlement intention of the new and old generations.

Variable	New generation			Old generation		
	Coefficient	Standard error	*P-*value	Coefficient	Standard error	*P-*value
Demographics characteristics						
Gender (reference group: female)						
Male	−0.158	0.175	0.367	−0.054	0.172	0.752
Education level (reference group: primary school and below)						
Junior high school, senior high school, technical secondary school	0.043	0.313	0.890	0.089	0.168	0.596
Bachelor degree or above	1.008*	0.400	0.012	2.033*	0.946	0.032
Father's household registration type (reference group: agricultural registered permanent residence)						
Non-agricultural registered permanent residence	−0.627	0.419	0.134	0.397	0.414	0.338
Political outlook (reference group: non-party members)						
Membership of the Communist Party of China	−0.045	0.376	0.905	0.236	0.644	0.714
Marital status (reference group: unmarried/divorced/widowed)						
Married	−0.067	0.202	0.741	0.274	0.501	0.584
Number of family members	0.122**	0.044	0.005	0.111*	0.048	0.022
Self-rated health	−0.146	0.120	0.225	−0.125	0.090	0.163
Annual household income	0.000**	0.000	0.008	0.000*	0.000	0.060
Mobility						
Number of migrations	−0.066	0.060	0.269	−0.156**	0.059	0.009
Parents' mobility experience (reference group: none)						
Yes	−0.067	0.181	0.713	−0.432*	0.256	0.092
Reasons for mobility (reference group: work-study mobility)						
Matrimonial migration	0.176	0.295	0.550	0.711*	0.300	0.018
Demolition and moving flow	0.164	0.591	0.781	−0.449	0.523	0.391
Urban environment (built environment and social environment)						
Social environment						
Number of friends	0.026**	0.010	0.008	0.007	0.008	0.328
Community trust degree	0.002	0.109	0.984	0.095	0.087	0.273
Participation in community organization activities	0.030	0.063	0.634	−0.031	0.116	0.791
Community security	−0.032	0.020	0.114	0.146	0.020	0.468
Built environment						
Urbanization rate	−3.505	4.120	0.395	−9.860*	5.047	0.051
Population density	0.000	0.001	0.932	0.001	0.001	0.132
POI number per 10,000 people	0.001	0.001	0.184	0.001	0.001	0.335
Land use situation	−2.423	5.539	0.662	−10.078*	5.445	0.064
Per capita GDP	0.000	0.000	0.753	0.000*	0.000	0.047
Green coverage rate	−1.143	10.225	0.911	19.682*	9.599	0.040
Annual average concentration of PM2.5	−0.065	0.062	0.295	0.094	0.066	0.154
Number of hospitals and health clinics	0.005*	0.003	0.094	−0.004	0.003	0.121
Constant	7.231	4.376	0.098	−3.947	3.665	0.282

***p < 0.01, **p < 0.05, *p < 0.1.

However, the new generation has a stronger willingness to improve their own income level, pays little attention to urbanization level and land-use intensity, and the negative impact on their settlement intention is not obvious. In terms of mobility, the number of migrations and marriage migration had a significant impact on their settlement intention. Compared with the new generation, the old generation has more capital for migration activities, and their settlement intention is lower when there are more migration times and a richer migration experience. The old generation has had more time to form families. Marriage maintains family stability, and its positive effect on settlement intention is much more obvious than that of the new generation. There was no significant relationship between marriage migration and the new generation’s settlement intention, which is related to the new generation’s emphasis on the realization of self-worth and their lack of family values (36).

For the new generation, the number of hospitals and health centers is more related to the built environment, which is positively correlated with their settlement intention. This may be related to the promotion of healthcare awareness among the new generation, focusing on the construction of surrounding medical service facilities (64). In addition, the number of friends was a significant factor affecting the new generation’s settlement intention. Compared to the migration of the old generation due to rural affinity and kinship, the new generation has richer reasons for migration; their social network is simpler when they first flow into the city, and they often expect a higher sense of social identity.

In summary, the differences between old and new generations are reflected in three aspects: built environment, mobility, and social environment. The new generation pays more attention to the degree of social integration when flowing into cities and people with more developed social networks tend to stay in local areas. Sociologist Park believed that interpersonal networks can reduce the cost and risk of migrants’ mobility and increase their sense of security and belonging (65), which is fully reflected in the settlement intention of the new generation. When studying the influence of human capital on the social integration of the migrants in China, we mainly discuss the influence of human capital obtained before mobility, that is, education level, on social integration. Some articles point out that human capital obtained after mobility, that is, skills training and work experience, also play an important role in social integration (66). Generally, the new generation of migrants has accumulated less human capital, has lower social integration, and they pay more attention to their social identity. Social environment significantly influences their settlement intention. The old generation pays more attention to the characteristics of mobility and built environment. Because of their age and concerns, one of the purposes of promoting the mobility of the old generation is usually to experience “living in peace.” With the gradual completion of family formation, accompanying family members also have higher settlement intentions. Existing research has found that green spaces can enhance residents’ local attachment and settlement intention (67). Owing to physical function, health status, and family factors, the requirements of the old generation for green spaces are greater.

### 5.3 The common between the new and old generations

The model results show that the influence of settlement intention varies from generation to generation, but also has some similar influencing factors. Education level, number of family members, and family income had a significant influence on both generations. Education has different influences on the settlement intentions of the two generations, but it is still one of the factors that determines whether they want to stay in the local area. The old generation has richer social experience, is more sensitive to future income levels represented by education levels than the new generation, and is more willing to leave family members in areas with better education levels (28). Therefore, compared to the new generation, the old generation’s settlement intention will be more affected by education level. An increase in the number of family members improves the settlement intention of the two generations, and the establishment of kinship in the inflow area can improve psychological support for the migrants and settlement intention of the two generations (50, 58). For both generations, family income level is very important, as it is necessary to maintain their survival and pursue a better life. In the context of the accelerating growth of urban housing prices and increasing consumption levels, people have greater demand for a better life, and a higher income level can provide a better and more comfortable living environment, which is very attractive to the migrants. Although the two generations grew up in different social backgrounds, higher income and family composition were still the driving forces for the migrants to continue migrating to cities (68), and these increases undoubtedly enhance the migrants’ intention to settle in cities.

## 6 Discussion and conclusion

Given the background of people-oriented new urbanization in China, the settlement intention of the migrants has important research significance. The new and old generations of the migrants have different group characteristics, and attach different meanings to mobility. The new generation has gradually become the backbone and creative class to promote social development (69), and expects to realize its ideals in inflow places, while the migrants of the old generation is still an important group in China’s labor market. Against this background, this study investigates the migrants in the Pearl River Delta region, as well as the influence of demographics characteristics, mobility and urban environment on settlement intention, and compares the new with the old generation. The study obtains the following findings:

First, the migrants’ settlement intention is influenced by the built environment, mobility, social environment, and demographic characteristics. In terms of the built environment, urbanization rate, population density, land use, and green coverage rate have a significant impact on their settlement intention. The number of migrations has a significant impact on the migrants’ settlement intention, and marriage mobility is associated with stronger settlement intention than other mobility modes. Social environment reflects the important influence of social networks and human capital on settlement intention. The more friends, the stronger the settlement intention of the migrants. Educational back-ground, family income, and number of family members are important factors affecting the settlement intention of both generations. Second, there are differences in the built environment, social environment, and demographic characteristics between the new and old generations. The new generation of migrants generally has a higher settlement intention. For the new generation, demographics and social environment have a significant impact on their settlement intention. In terms of social environment, having more friends and a higher degree of trust had a positive impact on the new generation’s settlement intention. The flow and built environment of the old generation differed from those of the new generation. Among the mobility, the old generation’s settlement intention was influenced more by the number of migrations, parents’ migration experiences, and personal migration experiences due to marriage. Among the built environment, the settlement intention of the old generation is more sensitive to the green coverage rate. From the differences between the two, we can see the focus of the new and old generations and that the new generation has a higher pursuit of social identity and economic strength; the old generation is more influenced by residence time, family concept, and physical and mental health. Third, the settlement intention of the new and old generations was positively influenced by their educational level and family members.

By focusing on the complex mechanisms behind the migrants and their intergenerational settlement intention, our empirical analysis expands the perspective of the urban built environment, which helps optimize the relevant decision-making for high-quality urbanization and community governance in the Pearl River Delta region. First, the living environment had a significant impact on the migrants’ settlement intention. The improvement of space quality helps to enhance the migrants’ sense of belonging, acquisition, and identification with the inflow location, especially the older generation of migrants who are more sensitive to environmental quality and have higher requirements for livability and can enhance their settlement intention. While promoting urban economic development, the government should improve the livable level of cities. Future urban construction should focus on improving the building environment quality of central cities, issuing more scientific urban planning policies, rationally utilizing urban land, and improving the comprehensive carrying capacity of cities. At the same time, we should pay special attention to the construction of urban green space, optimize the green environments around aging communities, improve the availability of green space environments, improve the quality of the urban ecological environment, and improve the living level of cities.

Second, economic opportunities remain an important determinant of the migrants’ settlement intention. The new generation of migrants is more eager to improve their income level. It is necessary for the government to introduce personalized and targeted talent attraction policies, and provide richer and more diversified economic opportunities for the new generation of migrants. And provide inclusive policy support in housing, employment, entrepreneurship, and social welfare, including controlling the increase of urban housing prices, providing more accessible housing for migrants, introducing preferential loan policies for entrepreneurship, and providing fair social welfare. Reduce the life pressure of the new generation of migrants, provide more development space, improve their income level, and enhance their settlement intention.

Third, the migrants are paying increasing attention to guarantees of public services in urban life. Improving service capacity and group fairness of public service facilities is a key to solving the problem that cities cannot retain people. Efforts should be made to resolve barriers to the regional sharing of public services and social security information. Lower the educational entry threshold for migrant children in local cities, increase the supply of local degrees, and provide more fair educational resources for migrants. The enhancement of the new generation of migrants’ awareness of medical care requires cities to improve the medical and health service system, increase the construction of primary medical and health facilities, and clarify the reimbursement process of medical insurance for migrants in different places to improve the convenience of migrants’ access to medical services.

Fourthly, social integration plays a non-negligible role in migrants’ settlement intentions. Especially for the new generation of migrants, the degree of social integration in the inflow area has a positive impact on their settlement intention. The community should regularly organize social and cultural activities, carry out community education and community mobilization, increase the interaction among community residents, enrich migrants’ social networks in the community, and promote migrants’ integration into the community. At the same time, it provides migrants with equal opportunities to participate in community elections and policy decisions and enjoy community services and social services fairly. And in the context of aging, the social integration of the older generation of migrants is also very important. More attention should be paid to the strategic change of community governance in the context of aging, especially the social integration of the older generation of migrants and the children who move with them. It is necessary to create a developed, inclusive, and equal urban atmosphere, provide a more beneficial social environment for migrants to integrate into urban society, and promote the promotion of migrants’ settlement intention.

This study has some limitations. First, the data selected in this study is cross-sectional, and there may be missing variables or unobservable differences between individuals in the statistical collection of cross-sectional data. Secondly, the database used in this study is the static data in 2016. Although it has research significance, there is still a certain lag with the current society. Moreover, with the acceleration of urbanization in the Pearl River Delta region, the settlement intention of migrants may change. If the survey of migrants can be tracked, it will be helpful to continuously observe whether the settlement intention of migrants will change with time, thus further improving the quality of research results. At the same time, the COVID-19 pandemic has a momentous influence on the migration and settlement of Chinese and even the world, and future research work should also pay attention to the different influences of the COVID-19 pandemic on the settlement willingness of migrants. The intergenerational differences in the factors affecting the settlement intention of migrants show that the settlement intention of migrants evolves dynamically with the development of society. In the new urbanization process of regional coordinated development in the future, it is an important issue to attract migrants and make them feel a sense of belonging to the flowing cities.

## Data availability statement

The original contributions presented in the study are included in the article/supplementary material, further inquiries can be directed to the corresponding author.

## Author contributions

XL: Conceptualization, Data curation, Writing – original draft. QL: Conceptualization, Data curation, Methodology, Writing – original draft. WZ: Conceptualization, Formal analysis, Writing – original draft. RW: Funding acquisition, Resources, Supervision, Writing – original draft.
