# Corrigendum: How does mobility and urban environment affect the migrants' settlement intention? A perspective from the intergenerational differences

**DOI:** 10.3389/fpubh.2024.1528911

**Published:** 2025-01-06

**Authors:** Xiaoxiang Liang, Qingyin Li, Wen Zuo, Rong Wu

**Affiliations:** ^1^Baiyun Branch of Guangzhou Urban Planning & Design Survey Research Institute, Guangzhou, China; ^2^School of Architecture and Urban Planning, Guangdong University of Technology, Guangzhou, China

**Keywords:** migrants, settlement intention, new generation, urban environment, mobility

In the published article, there was an ambiguity in the Acknowledgment section. Yawen Chen, Shiyan Hong, Yanrong Qiu, Yueyi, Huang and Jingpeng Luo from Guangdong University of Technology were not mentioned in the Acknowledgments section.

The Acknowledgments section previously stated: “We sincerely thank the couples who participated in the study”.

The corrected Acknowledgment statement appears below:

